# Monitoring Compliance and Acceptability of Intermittent Preventive Treatment of Malaria Using Sulfadoxine Pyrimethamine after Ten Years of Implementation in Tanzania

**DOI:** 10.1155/2017/9761289

**Published:** 2017-03-30

**Authors:** Mdetele B. Ayubu, Winifrida B. Kidima

**Affiliations:** College of Natural and Applied Sciences, University of Dar es Salaam, P.O. Box 35064, Dar es Salaam, Tanzania

## Abstract

Intermittent preventive treatment using SP (IPTp-SP) is still a superior interventional approach to control malaria during pregnancy. However its rate of use has gone down tremendously in malaria endemic areas. This study forms part of a larger study aimed at monitoring the compliance of IPTp-SP policy in malaria endemic areas of Tanzania. Two cross-sectional studies were conducted in Dar es Salaam and Njombe Regions of Tanzania. Overall, 540 pregnant women and 21 healthcare workers were interviewed using structured questionnaires. This study revealed that 63% of women were not willing to take SP during pregnancy while 91% would only take it if they tested positive for malaria during antennal visits. 63% of the interviewed women did not know the recommended dose of SP required during pregnancy, despite the fact that 82% of the women were aware of the adverse effect of malaria during pregnancy. It was found out that 54% of pregnant women (30–40 weeks) took single dose, 34% took two doses, and 16% did not take SP at the time of interview. It was also found that SP was not administered under direct observed therapy in 86% of women. There was no significant relationship between number of doses received by pregnant women and antenatal clinic (ANC) start date (*r*^2^ = 0.0033, 95% CI (−0.016 to 0.034)). However positive correlation between drug uptake and drug availability was revealed (*p* = 0.0001). Knowledge on adverse effects of placental malaria among pregnant women was significantly associated with drug uptake (OR 11.81, 95% CI (5.755–24.23), *p* = 0.0001). We conclude that unavailability of drugs in ANC is the major reason hindering the implementation of IPTp-SP.

## 1. Introduction

Intermittent preventive treatment using Sulphadoxine Pyrimethamine (IPTp-SP) has proven to be effective in reducing the burden of malaria during pregnancy [1–5]. SP is still a prophylactic drug of choice against malaria during pregnancy. Although 2 doses of SP have been the standard for IPTp-SP, 3 doses might be more effective in reducing burden of poor pregnancy outcome due to malaria [6]. The World Health Organization recommended that women in malaria endemic areas should take a monthly dose from second trimesters till term instead of the initially recommended 2 doses [7]. Tanzania adopted IPTp-SP program since year 2000 [8] with total coverage of about 32% [9]. According to Demographic Health Surveillance survey IPTp-SP use has been increasing with percentages of pregnant women using more than three doses increasing from 3% in 2004 to 8% in 2015 [10]. Despite the evidence of the effectiveness of IPTp strategy using SP in reducing the adverse effects of malaria during pregnancy, IPTp-SP compliance and therefore the uptake and coverage have been reported to be low in Tanzania [11–13]. Low uptake of SP may increase rate of placental parasitaemia and hence rates of miscarriage, stillbirth, and delivering of low birth babies. WHO recommend regular monitoring of IPTp-SP among women in malaria endemic areas. Therefore there is a need to constantly monitor factors that are associated with low uptake of SP among the women in endemic areas.

Apart from IPTp-SP, insecticide treated nets (ITN) have been utilized on large scale in Tanzania and in selected places indoor residual spraying is being utilized to reduce human- mosquito contact. The use of ITN has accounted for about 50% reduction of malaria cases [14, 15]. However residual malaria which is the result of mosquito behavioral change [16, 17] together with increasing mosquitoes insecticide resistance has put pregnant women at high risk to acquire malaria despite the use of ITN. Following that, the use of IPTp-SP remains critical in minimizing effect of placental malaria in endemic areas.

In this study we aimed at investigating knowledge, acceptability of IPTp-SP, and factors that affect compliance of IPTp-SP in Tanzanian after more than ten years of adaptation. Specifically we assessed the proportion of women who were aware of the adverse effect of placental malaria, assessed availability of SP in public antenatal clinics, and outlined factors that are associated with low uptake of IPTp-SP by pregnant women. We also assessed the willingness of pregnant women in testing for malaria during regular ANC visits. Information from this study will help to improve strategies to increase access of SP to pregnant women.

## 2. Methodology

### 2.1. Study Area and Sampling Design

This study involved two cross-sectional surveys carried out in February–April 2015 and February–June 2016 in four districts: three (Ilala, Temeke, and Kinondoni) from Dar es Salaam urban, eastern coast of Tanzania, and one (Njombe District) from Iringa. Dar es Salaam (6.7924°S, 39.2083°E) is bounded by the Indian Ocean on the east and by the Coast Region on the other three sides. The area experiences tropical climatic conditions, characterized by hot and humid weather throughout much of the year. Annual rainfall is approximately 1,100 mm. Njombe is located in Iringa region, in the southern part of Tanzania. It is centered around latitude of 7.77°S and longitude of 35.69°E, 1,581 meters elevation above sea level. Both Dar es Salaam and Iringa are malaria hyperendemic areas. We selected thirteen different antenatal clinics purposefully based on ANC availability.

A researcher was given an opportunity to describe the study to pregnant women attending ANC. A questionnaire with closed and open ended questions was administered to 50% of randomly selected consenting pregnant women. Information included social and demographic information, availability of SP at the clinic, ANC start date, use of ITN, gravidity, education level, malaria positivity during pregnancy, awareness of IPTp-SP program, acceptability/willingness to take SP as an malaria interventional program during pregnancy, the mode in which SP was administered (utilization of DOT), number of doses of SP received, availability of SP at the clinic, side effect observed, knowledge on adverse effect of malaria during pregnancy, recommended dosage of SP, preference to test for malaria at ANC, and participants view on improvement of IPTp-SP.

A health provider questionnaire was also administered to enquire about availability of SP at the clinic and reasons for lower uptake of IPTp-SP among pregnant women, provision of information about the IPTp-SP program, and other factors affecting uptake of SP compliance on IPTp-SP.

Research clearance for the project was obtained from the Commission for Science and Technology Tanzania (NDC/P.20/2/VOLIII/14) through the University of Dar es Salaam Vice Chancellor office. Permission to conduct this study in three districts in Dar es Salaam and Njombe was obtained from the Regional Administrative Secretary (RAS), District Medical Officer in charge of the respective facilities. Written consent from the study participant was obtained from 540 pregnant women and 21 healthcare workers.

### 2.2. Sample Size Calculation

The sample size calculation was based on the formula by Pfeiffer (2002) = *Z*^2^*P*(1 − *P*)/*d*^2^, where *P* is the rate of optimal uptake of IPTp-SP in Tanzania which is estimated to be 43% [11], *Z* stands for a standard normal variate at 5 (1.96), and the absolute error or precision *d* is 0.05. The minimum sample size obtained was 376. This was distributed within the two study sites. All health workers who consented were interviewed in this study.

### 2.3. Data Analysis

Data analysis was performed using Graph Prism version 5. Fisher exact test was used to test for association between knowledge on impact of placental malaria and drug uptake.

Linear regression was used to analyze relationship between number of doses received and ANC start date. The association between drug uptake and drug availability of SP at ANC was analyzed using Spearman's rank correlation test.

## 3. Results

### 3.1. Characteristics of Study Subjects

Five hundred and forty pregnant women and 21 health workers were successfully interviewed in Dar es Salaam and Njombe. The mean age range for pregnant women interviewed was 27 (15–42) years. The majority (39%) of women were multigravidae (Table 1). 22% of women were primigravidae. Forty-seven percent of pregnant women had been to secondary schools. Only 14% of pregnant women reported having tested positive for malaria during the time of pregnancy. All women interviewed reported to have been using insecticide treated nets.

### 3.2. Knowledge of the Adverse Effect of Placental Malaria on Pregnancy Outcomes and the Recommended Dose of SP in IPTp

**Figure 1:**
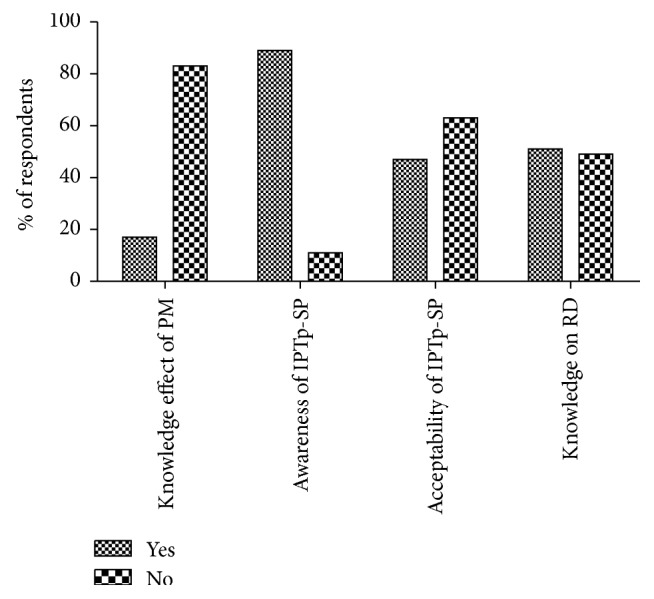
Percentage respondents (pregnant women) on knowledge of placental malaria, awareness, acceptability, and recommended dose of IPTp-SP among pregnant women in Dar es Salaam and Njombe. RD: recommended dose; PM: placental malaria (*n* = 540).

Most of the interviewed women were aware of IPTp-SP as an intervention to control malaria during pregnancy (Figure 1). However, about 49% of women who were aware of IPTP- SP did not know the recommended dosage required during pregnancy to control malaria (Figure 1). In addition to the knowledge on SP dosage, a large proportion (451/540) of interviewed women did not know the effect of malaria during pregnancy (Figure 1). Effect of malaria during pregnancy outlined by 89/540 women included preterm delivery (69%, *n* = 63), maternal and neonatal deaths (13%, *n* = 15), and congenital malaria (17%, *n* = 11). Using Contingency Tables it was found that knowledge on adverse effects of placental malaria among pregnant women was associated with drug uptake (odds ratio (OR) = 11.81, 95% CI (5.755–24.23), *p* = 0.0001, Fisher exact test).

### 3.3. IPTp-SP Acceptability and Compliance

Contrary to what we had predicted, it was found that a large proportion (63%, *n* = 340) of women were not willing to take SP during pregnancy, 9% (*n* = 31) citing fear of side effects and 91% (*n* = 309) with no clear reason(s). In addition it was found that about 91% (*n* = 491) of women preferred to test for malaria before agreeing to take SP. Only third-trimester pregnant women (weeks 28–36) were interviewed on SP dosage received at the time of interview. In view of this only 138 responses were analyzed. About 54% (*n* = 74) of women took single dose, 34% (*n* = 50) took two doses, and 16% (*n* = 22) did not take SP at the 3rd trimester. Ninety-two percent (*n* = 68) of pregnant women who received a single dose declared that they were not given SP on follow-up visits. No significant relationship was found between ANC start date and dosage received (Pearson's *r*^2^ = 0.003261, 95% CI (−0.01639 to 0.03376)). However, there was a positive correlation between drug uptake and drug availability of SP at ANC (Spearman *r* = 0.67, *p* = 0.0001, 95% CI (0.54 to 0.77)). We also asked whether the SP were administered by directly observed therapy (DOT) as recommended by WHO. Overall we found DOT was not observed in 86% (314/364) of all pregnant women who were given SP.

### 3.4. Health Workers Responses

A total of 21 healthcare workers from different antenatal clinics were interviewed on factors affecting uptake of SP by pregnant women. 100% confirmed provision of information about IPTp-SP to pregnant women at the clinic. 95% (*n* = 20) of health workers declared that SP is not always available at the clinics. We found SP was not available in 77% of antenatal clinics at the time of interview. Other reasons for low uptake of SP outlined included late attendance to antenatal clinics by some of pregnant women.

## 4. Discussion

In the present study we found that, despite high level of awareness and knowledge about IPTp-SP, the acceptability and uptake of SP among pregnant women are still low. Additionally this study establishes a link between low supply of SP in ANC, knowledge about impact of placental malaria by pregnant women, and uptake of SP. These results support the observations by Marchant et al., 2008, Mpogoro et al., 2014, and Mubyazi and Bloch, 2014, who observed SP unavailability and low uptake of SP among women in Tanzania [13, 18, 19]. Low uptake of SP may increase risk of malaria parasitaemia and therefore placental malaria prevalence among pregnant women in endemic areas.

Studies have shown that uptake of IPTp-SP has been increasing with percentages of pregnant women taking more than 3 doses rising from 3% in 2000 to 8% in 2015 [10]. Majority of the third-trimester women in our study received a single dose of SP at 28 to 40 weeks of pregnancy. Mubyazi and colleague, 2014, observed that delivery and uptake of IPTp-SP were affected by late ANC attendance [13]. Surprisingly, we did not find a relationship between ANC start date and dosage of IPTp-SP received by pregnant women. However an association between availability of SP in clinics and uptake was observed in this study. Studies have shown that SP is not always available in clinics in other malaria endemic areas such as Nigeria [13, 20], suggesting a major barrier to IPTp-SP implementation. Unavailability of SP in clinics may result in women taking suboptimal prophylactic doses. Multiple doses of SP have been shown to improve clinical outcomes in pregnant women in malaria endemic areas. For example, two or more doses have been shown to be effective in reducing prevalence of malaria parasitaemia [4] and therefore improve pregnancy outcomes. Constant supply line of SP drugs remains critical in facilitating uptake of SP among pregnant women in malaria endemic areas and therefore a major challenge to be addressed.

Although DOT has been shown to improve compliances to IPTp-SP, its utilization at ANCs in many malaria settings has been shown to be challenging [21]. DOT was not observed in majority of women receiving SP in this study. In spite of high level of knowledge about IPTp-SP as an intervention among the women in this study, high refusal rates were observed. In Malawi limited understanding of IPTp-SP among pregnant women affected uptake [22], emphasizing the need to provide knowledge on placental malaria and IPTp-SP program to pregnant women in endemic areas.

IPTp-SP is still a superior interventional program for controlling malaria [23] among pregnant women. A randomized control trial of IPTp-SP versus IPTp-Artemisinin Lumefantrine (AL) on women in low malaria transmission areas indicated that SP was superior in reducing malaria parasitaemia and performed equally well as IPTp-AL in improving pregnancy outcomes (reviewed in [23]). Furthermore, studies by Desai et al. 2015 reported that intermittent screening and treatment in pregnancy have no value addition compared to the use of SP alone insisting superiority of IPTp-SP during pregnancy [24]. In the current study many women preferred to test for malaria before the uptake of SP suggesting more provision of information, education, and communication about the benefits of IPTp-SP uptake.

Unfortunately we were unable to evaluate from this data the effect of low dosage of SP on pregnant outcomes among the pregnant women studied. Other limitations in our study include small number of third trimester women analyzed and the randomization procedure that may have led to sampling bias. Notwithstanding its limitation this study showed higher refusal rates of IPTp-SP among studied women. Higher refusal rates against IPTp-SP signal poor delivery of knowledge on IPTp-SP among women and calls for alternative IPTp-SP-knowledge-delivery mechanisms. We recommend that SP should be available in ANCs for successful scale-up of IPTp-SP program.

## Figures and Tables

**Table 1:** Characteristics of study subjects.

Mean age in years (range)	27 (15–42)
*Gravid*	Proportions% (*n*)
Primigravidae	29 (157)
Secundigravidae	32 (175)
Multigravidae	39 (208)
*Weeks of pregnancy*	
First trimester	16 (85)
Second trimester	59 (317)
Third trimester	25 (138)
*Level of education *	
No school	12 (64)
Primary education	24 (130)
Secondary education	47 (251)
Tertiary education	16 (88)
No response	1 (7)
*Malaria positive during pregnancy (self-reported cases) *	
First-trimester women, *N* = 85	5 (28)
Second-trimester women, *N* = 317	3 (18)
Third-trimester women, *N* = 138	6 (32)
*ITN usage*	
Primigravidae	29 (157)
Secundigravidae	32 (175)
Multigravidae	39 (208)
